# Magnetic Properties of Ferromagnetic Particles under Alternating Magnetic Fields: Focus on Particle Detection Sensor Applications

**DOI:** 10.3390/s18124144

**Published:** 2018-11-26

**Authors:** Ran Jia, Biao Ma, Changsong Zheng, Liyong Wang, Xin Ba, Qiu Du, Kai Wang

**Affiliations:** 1 School of Mechanical Engineering, Beijing Institute of Technology, No. 5 South Zhongguancun Street HaiDian District, Beijing 100081, China; jiaran89@126.com (R.J.); mabiao@bit.edu.cn (B.M.); baxin0802@163.com (X.B.); duqiu@bit.edu.cn (Q.D.); 2120160388@bit.edu.cn (K.W.); 2 The Ministry of Education Key Laboratory of Modern Measurement and Control Technology, Beijing Information Science and Technology University, No. 12 Xiaoying East Street, HaiDian District, Beijing 100192, China; wangliyong@bistu.edu.cn; 3 Faculty of Engineering and Information, University of Technology Sydney, Ultimo, NSW 2007, Australia

**Keywords:** magnetic properties, wear particle, sensors

## Abstract

The electromagnetic wear particles detection sensor has been widely studied due to its ability to monitor the wear status of equipment in real time. To precisely estimate the change of the magnetic energy of the sensor coil caused by the wear particles, the magnetic property models of wear particles under the alternating magnetic field was established. The models consider the hysteresis effect and the eddy current effect of the wear particles. The analysis and experimental results show that with the increase of the effective field frequency, the change of the magnetic energy caused by the wear particles gradually decrease, which makes the induced electromotive force output by the sensor reduce with the decrease of the particle speed, so a signal compensation method is presented to obtain a unified signal when the same wear particle passing through the sensor in different speeds. The magnetic coupling effect between the two adjacent wear particles is analyzed. The result illustrates that the change of the magnetic energy caused by the dual wear particles system is larger than the sum of the energy variation caused by two independent wear particles, and with the increase of the interparticle distance, the magnetic coupling effect gradually weakens and disappears.

## 1. Introduction

Wear is one of the main reasons that cause mechanical equipment failures, and numerous wear particles are produced during the device running process [1]. These particles, as products of abrasion, may dramatically exacerbate the wear state of machinery. Generally, the debris material can roughly indicate the wear position in machinery and a sudden increase in the number of large wear particles in lubricating oil demonstrates latent or immediate faults [2,3]. Therefore, the wear debris monitoring technology is used to provide early failure prediction of mechanical equipment. Until recently, the offline wear particles detection methods are still commonly used for their preferable detection effect, though they are usually high cost, low efficiency and require a complicated setup. Over the past decades, there has been a growing need for online wear monitoring [4,5] and researchers have studied different kinds of online wear particle detection sensors based on optics, ultrasonic, capacitance and electromagnetics to monitor the number and size of the wear particles in lubricating oil [6]. Among these sensors, the electromagnetic wear particle detection sensor has been widely studied due to its advantages of simple structure, good temperature stability and anti-interference ability [7].

For a better detection effect, the electromagnetic wear particles detection sensors with different structures including single-coil [7,8], double-coil [9,10], parallel three-coil [11,12] and flat spiral coil [13,14] were proposed, and the sensitivity of the sensors is still one of the main reasons restricting their broader application. To improve the sensitivity of the sensor, Hong et al. [14] proposed a radial inductive debris detection sensor with a C-type iron core. This kind of core structure increases the magnetic field uniformity in the detection domain and improves the consistency of the detection results. Experimental results illustrated that 290 μm ferromagnetic particles can be successfully detected, while it’s hard to effectively detect non-ferromagnetic particles. In order to detect both ferromagnetic and non-ferromagnetic wear particles effectively, most electromagnetic particle detection sensors adopt a sinusoidal excitation signal to produce an alternating magnetic field in the sensor. Miller et al. [15] discussed a three-coil particle detection sensor (presented by GasTop Ltd., Ottawa, ON, Canada). To further improve the detectability for micro-debris of this kind of sensor, Li et al. [10] innovatively introduced the parallel LC resonance method into the single-coil sensor. Measurement results showed that the sensor is capable of detecting 20 μm ferromagnetic particles and 55 μm non-ferromagnetic particles in a 1 mm diameter channel. Recently, Zhu et al. [7] proposed a new method to heighten the detection effects of the dual-coil wear monitoring sensor by adding an iron core. They placed a pair of ferrite cores at the upper and bottom of the fluid channel, which enables this sensor to detect 11 μm and 50 μm ferromagnetic particles in a 1 mm and 7 mm fluidic pipes respectively. Another method to improve the sensitivity of the sensor is adopting the microchannel structure to decrease the distance between wear particles and the inner wall of the coil; however, this method greatly limits the maximum flow volume at the same time. Wu et al. [16] designed a microfluidic chip, based on the dual-coil detection principle, to detect wear particles. This sensor adopted a micro-fluid-channel with a diameter of 200 μm and an excitation signal with a frequency of 2 MHz, which enable the sensor to detect 5–10 μm ferromagnetic particles under the volume flow of 0.5 mL/min. However, this kind of sensor is difficult to adapt for practical applications because of such a small flow.

Although the various structural sensors are studied, the researches on the detection mechanism of wear particles are still extremely insufficient. He et al. [17] studied the magnetic field distribution of the sensor using the 3-D finite element method (FEM) and simulated the output signal of the sensor. Fan et al. [18] and Wu et al. [19] adopted the static magnetization model of the spherical particle to estimate the magnetic disturbance of the sensor caused by wear particle. This model ignores the hysteresis effect and eddy current effect of the wear particles under alternating magnetic field, and that can’t reflect the influence on detection effects by field frequency. Zhang et al. [20] and Fan et al. [21] studied the magnetic distribution around the wear particles under alternating magnetic field using the FEM. The results illustrate that with the increase of the field frequency, the eddy current effect leads to the uneven magnetic distribution in the wear particles. However, this model doesn’t consider the hysteresis effect and the magnetic energy loss of the ferromagnetic particles under alternating magnetic field, so it over-estimated the output signal of the sensor [22]. 

To solve the above problems and lay the foundation for parameter optimization of electromagnetic wear particle detection sensors, this paper builds a magnetic property model of the wear particles under the alternating magnetic field, which considers the hysteresis effect and the eddy current effect of the wear particle. Based on the model, the magnetic distribution of single wear particle and the magnetic coupling effect between two adjacent wear particles are analyzed. Meanwhile, the relationship between the sensor output signal and the magnetic disturbance caused by wear particles is illustrated. Besides that the hysteresis loss and the eddy current loss lead to the variation of the sensor signal as the change of the particle velocity, so a signal compensation method was presented to improve the accuracy and consistency of the test results.

## 2. Modeling of Magnetic Properties of Wear Particles

**Figure 1:**
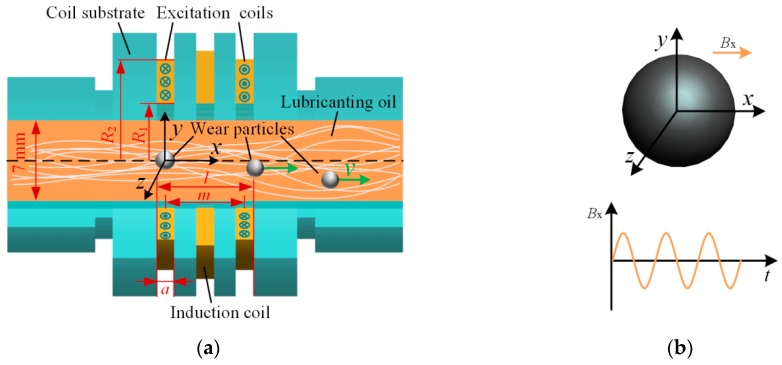
(**a**) The structure of the particle detection sensor; (**b**) The model of wear particle in the sensor.

The structure of a typical electromagnetic wear particles detection sensor is shown in Figure 1a. It is composed of two reverse excitation coils: an induction coil laying in the mid-plane of the sensor, and a coil substrate made of machinable ceramics (relative magnetic permeability $\mu_{r}\approx 1$). When sinusoidal currents are fed into the two excitation coils, two magnetic fields with opposite direction and same magnitude are induced by the two excitation coils, respectively, and counteract each other at the position of the induction coil. Under this condition, the magnetic flux in the induction coil is zero.

When wear particles pass through the sensor, the magnetic flux in the excitation coils changes, which leads to the flux variation of the induction coil and makes is generate an induced electro- motive force. 

In the debris detection process, wear particles of different shapes are generally described as an equivalent sphere by the equal volume method [10,14]. The model of a wear particle in the sensor is shown in Figure 1b. As our main objective is to study the magnetic properties of wear particles under the alternating magnetic field, it is assumed that wear particles move along the axis of the sensor. Therefore, the magnetic field around the particle can be regarded as a uniform alternating magnetic field. In the experiments, the structural limitation is used to guarantee the moving traces of particles (details are described in Section 4). 

The main factor that affects the output signal of the sensor is the change of the axial component of the sensor magnetic field. When no wear particles pass through the sensor, the axial magnetic flux density produced by single excitation coil ***B****_x_* can be described as [23]:

(1) $$\begin{array}{l} B_{x}(x,t)=\frac{\mu_{0}J_{\mathrm{coil}}}{2}(((x+\frac{a}{2})\ln\frac{R_{2}+\sqrt{{R_{2}}^{2}+{\left(x+a/2\right)}^{2}}}{R_{1}+\sqrt{{R_{1}}^{2}+{\left(x+a/2\right)}^{2}}})\\ -((x-\frac{a}{2})\ln\frac{R_{2}+\sqrt{{R_{2}}^{2}+{\left(x-a/2\right)}^{2}}}{R_{1}+\sqrt{{R_{1}}^{2}+{\left(x-a/2\right)}^{2}}})){\vec{e}}_{x} \end{array}$$

where *μ*_0_ = 4π × 10^−7^ is the permeability of vacuum, *J*_coil_ = *N*_e_*I*/(*a* × (*R*_2_−*R*_1_)) is the current density of the excitation oil, *N*_e_ = 140 is the turns of the excitation coil, *I* = 0.24sin(*ωt*) A is the drive current, *ω* is the angle frequency, *t* is the time, *a* = 2 mm is the width of the excitation coil, *R*_1_ = 10 mm and *R*_2_ = 15.6 mm are the inside and outside radius of the excitation coil, and ${\vec{e}}_{x}$ is the unit vector of the *x* axis.

The background magnetic flux density along the axis of the sensor ***B****_sx_* is a resultant magnetic flux density induced by the two excitation coils, and can be given by:

(2) $$B_{sx}(x,t)=B_{x}(x,t)-B_{x}(x+m,t)$$

where *m* = 9 mm is the center distance between the two excitation coils. As known from the calculation, the peak value of the magnetic flux density along the axis of the sensor is 2.5 mT.

The wear particles are affected by the alternating magnetic field of the sensor. Under this circumstance, the magnetic distribution in and around the wear particles satisfies the quasi-static condition as:

(3) $$\sigma \frac{\partial A}{\partial t}+\nabla \times ({\mu_{0}}^{-1}{\mu_{r}}^{-1}\nabla \times A)=0$$

where *μ*_r_ is the relative permeability of the material, *σ* is the conductivity of material, ***A*** is the vector magnetic potential, and ∇×() is the curl. Since the excitation source is sinusoidal, the vector magnetic potential ***A*** can be described as $A=Ae^{j\omega t}$, and $\frac{\partial A}{\partial t}=j\omega A$, where j is the imaginary unit.

Thus, the Equation (3) can be expressed as:

(4) $$j\omega \sigma A+\nabla \times ({\mu_{0}}^{-1}{\mu_{r}}^{-1}\nabla \times A)=0$$

According to the vector analysis theory, we can obtain that:

(5) $$\begin{array}{l} \nabla \times ({\mu_{0}}^{-1}{\mu_{r}}^{-1}\nabla \times A)=\frac{1}{\mu }[(\frac{\partial^{2}A_{y}}{\partial x\partial y}-\frac{\partial^{2}A_{x}}{\partial y^{2}})-(\frac{\partial^{2}A_{x}}{\partial z^{2}}-\frac{\partial^{2}A_{z}}{\partial x\partial z})]{\vec{e}}_{x}\\ +\frac{1}{\mu }[(\frac{\partial^{2}A_{z}}{\partial z\partial y}-\frac{\partial^{2}A_{y}}{\partial z^{2}})-(\frac{\partial^{2}A_{y}}{\partial x^{2}}-\frac{\partial^{2}A_{x}}{\partial x\partial y})]{\vec{e}}_{y}\\ +\frac{1}{\mu }[(\frac{\partial^{2}A_{x}}{\partial z\partial x}-\frac{\partial^{2}A_{z}}{\partial x^{2}})-(\frac{\partial^{2}A_{z}}{\partial y^{2}}-\frac{\partial^{2}A_{y}}{\partial z\partial y})]{\vec{e}}_{z} \end{array}$$

where *μ* is the permeability of the material, *A_x_*, *A_y_* and *A_z_* are the components of the vector magnetic potential along *x* direction, *y* direction and *z* direction respectively, while, ${\vec{e}}_{x}$,${\vec{e}}_{y}$ and ${\vec{e}}_{z}$ are the unit vectors of the *x* axis, *y* axis and *z* axis.

Therefore, the Equation (4) can be described as the equation set as:

(6) $$\begin{array}{l} j\omega \sigma A_{x}+\frac{1}{\mu }[(\frac{\partial^{2}A_{y}}{\partial x\partial y}-\frac{\partial^{2}A_{x}}{\partial y^{2}})-(\frac{\partial^{2}A_{x}}{\partial z^{2}}-\frac{\partial^{2}A_{z}}{\partial x\partial z})]=0\\ j\omega \sigma A_{y}+\frac{1}{\mu }[(\frac{\partial^{2}A_{z}}{\partial z\partial y}-\frac{\partial^{2}A_{y}}{\partial z^{2}})-(\frac{\partial^{2}A_{y}}{\partial x^{2}}-\frac{\partial^{2}A_{x}}{\partial x\partial y})]=0\\ j\omega \sigma A_{z}+\frac{1}{\mu }[(\frac{\partial^{2}A_{x}}{\partial z\partial x}-\frac{\partial^{2}A_{z}}{\partial x^{2}})-(\frac{\partial^{2}A_{z}}{\partial y^{2}}-\frac{\partial^{2}A_{y}}{\partial z\partial y})]=0 \end{array}$$

Lenz’s law illustrates that the direction of the eddy current is such that the magnetic field created by the eddy current opposes the background magnetic field. The magnetic field along the sensor axis oscillates also along the *x* axis.Therefore, based on the Ampère’s right-hand grip rule, the eddy current density in the particles satisfies *J*_x_ = 0. Under the sinusoidal alternating magnetic field $J_{x}=j\omega \sigma A_{x}$, the components of the vector magnetic potential around the spherical particles should satisfy:

(7) $$\begin{array}{l} A_{x}=0\\ \frac{\partial A_{x}}{\partial x}=\frac{\partial A_{y}}{\partial x}=\frac{\partial A_{z}}{\partial x}=0 \end{array}$$

**Figure 2:**
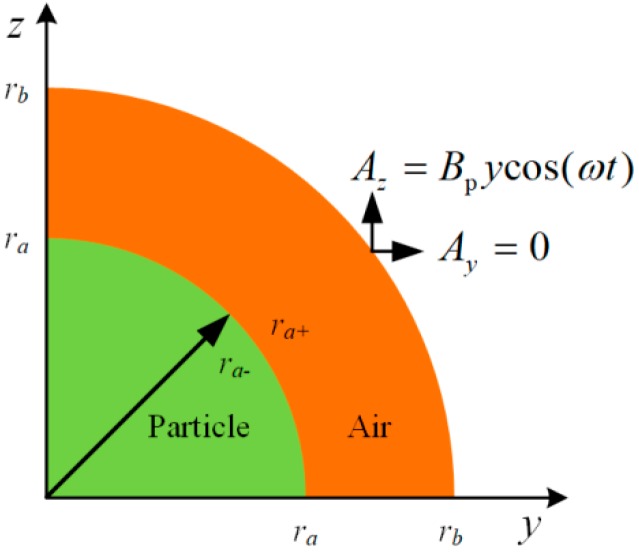
2D model of a 1/4 wear particle in the alternating magnetic field.

Thus, Equation (6) can be simplified to a set of two-dimensional equations as (8). The distribution of vector magnetic potential around a particle can be calculated by a 2D model as shown in Figure 2, where *r_a_* is the radius of the wear particle, and *r_b_* = 3 *r_a_* is the radius of the surrounding air which covers all the range of the magnetic variation caused by particles:

(8) $$\begin{array}{l} j\omega \sigma A_{y}+\frac{1}{\mu }(\frac{\partial^{2}A_{z}}{\partial z\partial y}-\frac{\partial^{2}A_{y}}{\partial z^{2}})=0\\ j\omega \sigma A_{z}-\frac{1}{\mu }(\frac{\partial^{2}A_{z}}{\partial y^{2}}-\frac{\partial^{2}A_{y}}{\partial z\partial y})=0 \end{array}$$

The magnetic distribution in and around the wear particles can be calculated by solving the above equations. During the calculation process, some boundary conditions and constitutive equations should be satisfied. According to the equation of B=∇×A, the vector magnetic potential of the background magnetic field ***A***_b_ can be described as:

(9) $$A_{b}=\left[\begin{array}{l} A_{x}\\ A_{y}\\ A_{z} \end{array}\right][{\vec{e}}_{x},{\vec{e}}_{y},{\vec{e}}_{z}]=\cos(\omega t)\left[\begin{array}{l} 0\\ 0\\ B_{p}\cdot y \end{array}\right][{\vec{e}}_{x},{\vec{e}}_{y},{\vec{e}}_{z}]$$

where $B_{p}=2.5\;\mathrm{mT}$ is the peak value of the background magnetic flux density.

At the outer boundary of the air, the magnetic vector potential equals to the background magnetic vector potential as illustrated in Equation (10). At the boundary between the wear particle and surrounding air, the distribution of magnetic vector potential is continuous and satisfies Equation (11):

(10) $$\underset{r\to r_{b}}{\lim}A=A_{b}$$

(11) $$\begin{array}{l} \underset{r\to r_{a-}}{\lim}A=\underset{r\to r_{a+}}{\lim}A\\ \underset{r\to r_{a-}}{\lim}\frac{\partial }{\partial r}(\frac{r}{\mu_{r}}A)=\underset{r\to r_{a+}}{\lim}\frac{\partial }{\partial r}(\frac{r}{\mu_{r}}A) \end{array}$$

In the wear particles and surrounding air, different constitutive equations are adopted. The relationship between the magnetic flux density ***B*** and magnetic field strength ***H*** in the air meets the Formula (12). Since both the hysteresis loss and eddy current loss exist in ferromagnetic wear particles under alternating magnetic field [24], the relative complex permeability is adopted as shown in Equation (13):

(12) $$B=\mu_{0}H$$

(13) $$\mu_{r}=\mu^{\prime }+\mu^{\prime \prime }=\frac{B}{\mu_{0}H}=\frac{B_{m}}{\mu_{0}H_{m}}(\cos\delta -j\sin\delta )$$

where $\mu^{\prime }$ and $\mu^{\prime \prime }$ are the real and imaginary components of the relative permeability respectively, $H_{m}$ and $B_{m}$ are the maximum values of *H* and *B*, and *δ* is the phase difference between *H* and *B*.

Therefore, the relationship between the magnetic flux density and magnetic field strength of the wear particles satisfy Equation (14). With this model, both the distribution of magnetic flux density in and around the wear particle and the magnetic disturbance of the sensor caused by wear particles can be calculated:

(14) $$B=\mu_{0}(\mu^{\prime }+j\mu^{\prime \prime })H$$

## 3. Simulation Analyses of the Magnetic Characteristics of the Wear Particle

### 3.1. Magnetic Distribution around Single-Wear Particle under Static Magnetic field

In mechanical systems, carbon steel is widely used in the vital transmission components, such as gear, shaft, flange, etc. These parts are the key targets of the wear monitoring, so the magnetic field distribution around the wear particle made of carbon steel is simulated by the proposed model. The simulation parameters including *B*_p_ = 2.5 mT, *μ*_r_ = 120, *ω* = 0, and *r_a_* = 0.125 mm are chosen to estimate the static magnetic properties of the ferromagnetic wear particle.

**Figure 3:**
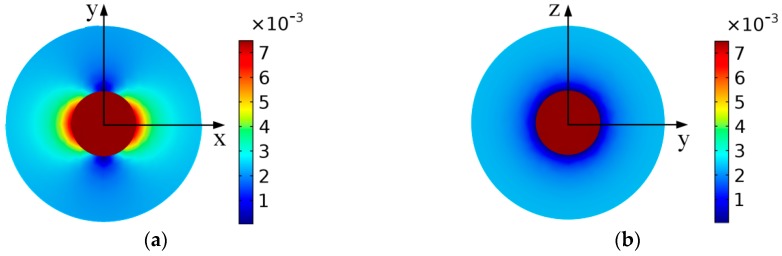
The distribution of the magnetic flux density around the particle. (**a**) *x*-*y* section of wear particle; (**b**) *y*-*z* section of wear particle.

**Figure 4:**
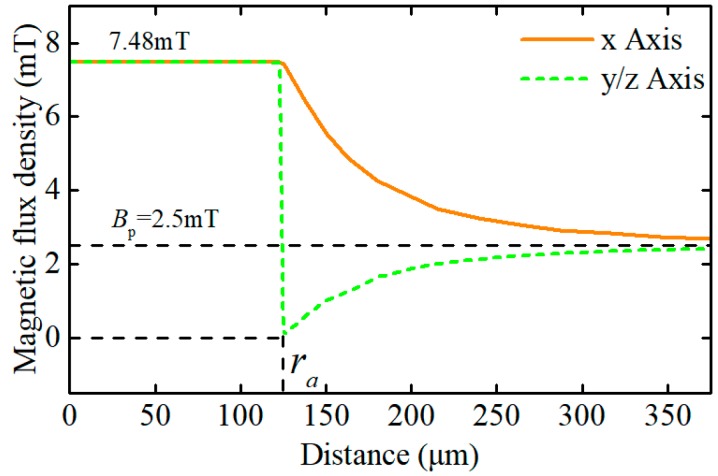
Magnetic flux density along the *x*, *y* and *z* axis.

The distributions of magnetic flux density in and around a wear particle under the static magnetic field are presented in Figure 3 and Figure 4. The simulation results illustrate that the magnetic field in the wear particle is evenly distributed, and the value of the magnetic flux density in the particle is *B*_inner_ *=* 7.48 mT. However, the magnetized wear particle disturbed the magnetic field of the surrounding air. More specifically, the magnetic flux density gradually declines from *B*_inner_ to *B*_p_ along the *x* axis, while along the *y* and *z* direction, the magnetic flux density near the particle-air interface decreases sharply from *B*_inner_ to 0, which may result from the magnetic cancellation between the inside and outside of the particle, and then gradually climbs back to *B*_p_. Therefore, for the purpose of calculating the output response of the sensor precisely, the magnetic distribution of both in and around the wear particle should be considered.

Under the static magnetic field, there exists an analytical solution about the magnetic distribution of the spherical ferromagnetic particle as shown in Equation (15), where ***B***_s_ is the saturated value of the magnetic flux density of the material. For the ferromagnetic particles, *μ*_r_ ≫ *μ*_0_ and the flux density of the wear particle in the sensor can’t reach the saturated value of the material, so the $B_{\mathrm{inner}}\approx 3B_{p}=75\;\mathrm{mT}$. It tells that the numerical solution agrees perfectly with the analytical solution, which validates the correctness of the proposed magnetic properties model of the wear particle:

(15) $$B_{\mathrm{inner}}=\{\begin{array}{l} \left(\frac{3\mu_{r}}{\mu_{r}+2}\right)B_{0},B_{0}<B_{s}\\ B_{0}+2B_{s},B_{0}>B_{s} \end{array}$$

### 3.2. Magnetic Distribution around a Single-Wear Particle under an Alternating Magnetic Field

For the electromagnetic wear particle detection sensors, the excitation frequencies range from 0 Hz to 3 MHz generally. It is the effective field frequency *f*_e_ that affecting the magnetic distribution of the wear particle rather than the excitation frequency of the sensor. The relationship between the real field frequency and the excitation frequency is shown in Equation (16):

(16) $$f_{e}=\frac{f_{0}}{f_{p}}$$

where *f*_0_ is the excitation frequency of the sensor, *f*_p_ = *v*/*l* is the frequency of particle passing through the sensor, *v* is the particle speed, *l* = 11 mm is outer distance between the excitation coils as shown in Figure 1a.

The excitation frequency of the sensor selected for the experiment is 100 kHz. To meet the flow requirement of lubricating oil (9 L/min), the maximum and the minimum flow speed are set to 4 m/s and 0.12 m/s, respectively. Therefore, the effective field frequency *f*_e_ ranges from 275 Hz to 9.2 kHz.

**Figure 5:**
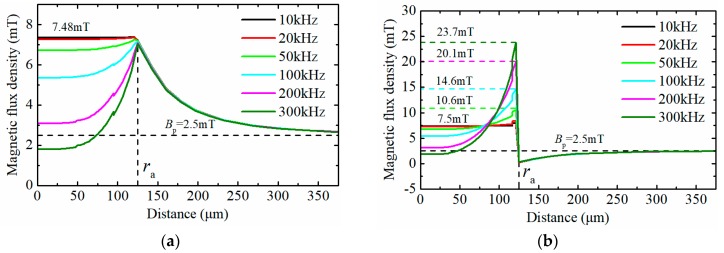
The distribution of magnetic flux density around the particle under different field frequencies. (**a**) The distribution of magnetic flux density along the *x* axis; (**b**) The distribution of magnetic flux density along the *y* or *z* axis.

With the increase of the effective field frequency, the eddy current effect in the wear particle becomes more and more significant [21]. The magnetic distribution around an iron particle under the alternating magnetic field is simulated based on the proposed model. Set the radius of the ferromagnetic particle to 0.125 mm. Figure 5 shows the flux density distribution in and around the wear particle under different effective field frequencies, when the hysteresis loss and eddy current loss are neglected ($\mu^{\prime }=120,\mu^{\prime \prime }=0$). It illustrates that when the effective field frequency is lower than 20 kHz, the eddy current effect in the particle can be neglected. As the effective field frequencies increase, the stronger eddy current effect causes inhomogeneity of magnetic distribution in the particle. The flux density at the center of the particle gradually becomes to 0, while the flux density at the surface of the particle gradually increases (23.7 mT when $f_{r}=300\;\mathrm{kHz}$), and the value of the flux density at the surface of the particle is much larger than the peak value of background flux desity (2.5 mT).

For ferromagnetic particles, the hysteresis effect and the eddy current effect are exist simultaneous under the alternating magnetic field, which leads to the hysteresis loss and eddy current loss in the wear particle. To study the influence on the magnetic disturbance of the sensor by these magnetic energy losses, we measured the relative complex permeability of the 45 steel (carbon steel) using the testing system for AC magnetic properties of soft materials (TD8120, Tunkia Co., Ltd, Changsha, China). The relative complex permeability of the material under the alternating magnetic field of different frequencies is illustrated in Table 1.

**Figure 6:**
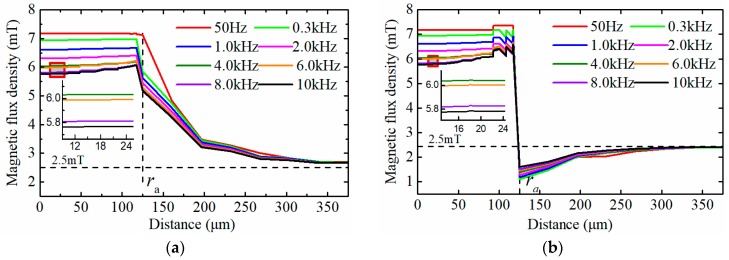
The distribution of the equivalent magnetic flux density under different effective field frequencies (**a**) The distribution of the equivalent magnetic flux density along the *x* axis; (**b**) The distribution of the equivalent magnetic flux density along the *y* or *z* axis.

The distribution of the equivalent magnetic flux density around the particle, when considering the hysteresis loss and eddy current loss, is shown in Figure 6. As shown in the figure, the magnetic flux density around the particle gradually decreases with the increase of the effective field frequency. The variation of the magnetic flux density around the wear particle leads to the change of the magnetic energy of the excitation coil which is the essential factor of influencing the induced electromotive force. The magnetic energy *W*_mf_ can be calculated by Equation (17), where *V* is the volume of the region where the magnetic field exists. The total variation of magnetic energy of the excitation coil $\Delta W_{c}$ is composed of the energy change in both the wear particle and the surrounding air, and it can be expressed as Equation (18).

(17) $$W_{\mathrm{mf}}=\frac{1}{2}\int \frac{B^{2}}{\mu }dV$$

(18) $$\Delta W_{c}=\Delta W_{p}+\Delta W_{a}=\frac{1}{2}\int \frac{\Delta B^{2}}{\mu^{\prime }\mu_{0}}dV_{p}+\frac{1}{2}\int \frac{\Delta B^{2}}{\mu_{0}}dV_{a}$$

where Δ*W*_p_ is the change of magnetic energy in the wear particle, Δ*W*_a_ is the change of magnetic energy in the surrounding air, Δ***B*** is the variation of the flux density, *V*_p_ and *V*_a_ are the volumes of the wear particle and surrounding air, and *μ*′ is the real component of the relative complex permeability of material.

**Figure 7:**
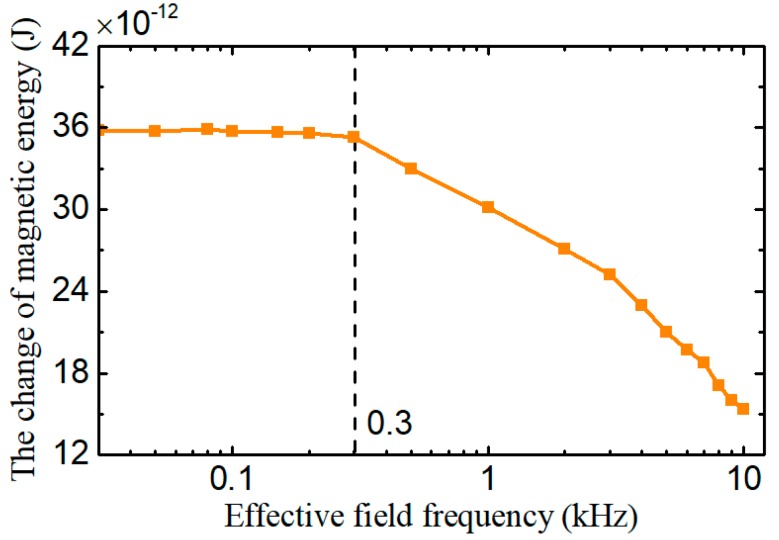
The change of magnetic energy stored in excitation coil under different effective field frequencies.

Based on the data of Figure 6, the total change of the magnetic energy of the excitation coil Δ*W*_c_ caused by a wear particle with the radius of 125 μm is calculated and depicted in Figure 7. It shows that when the effective field frequency is lower than 0.3 kHz, the total change of the magnetic energy of the excitation coil remains constant. However with further increase of the effective field frequency, the change of magnetic energy of excitation coil gradually declines. The Equation (16) indicates that the effective field frequency is inversely proportional to the particle speed. Therefore, Figure 7 means that when the particle speed is lower than 3.6 m/s (corresponding effective field frequency is 0.3 kHz), the induced electromotive force by the sensor reduces as the decrease of the particle speed.

### 3.3. Magnetic Distribution of Dual Wear Particles System

**Figure 8:**
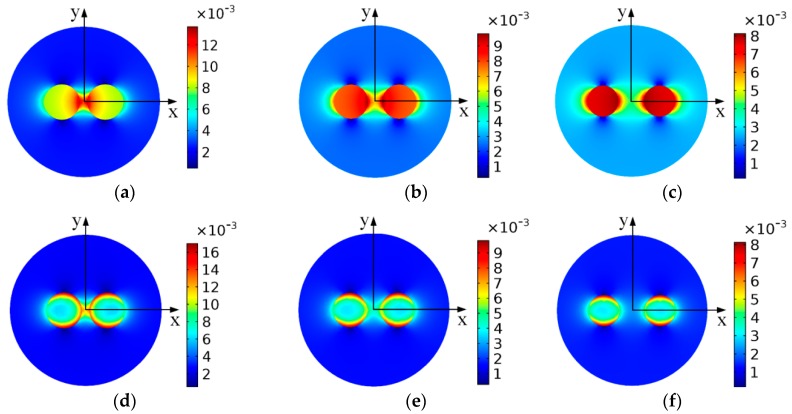
The flux density distribution of the dual wear particles system (**a**) *f*_e_ = 0 Hz, *l*_p_ = 0.4*r_a_*; (**b**) *f*_e_ = 0 Hz, *l*_p_ = 0.8 *r_a_*; (**c**) *f*_e_ = 0 Hz, *l*_p_ = 1.6 *r_a_*; (**d**) *f*_e_ = 100 kHz, *l*_p_ = 0.4 *r_a_*; (**e**) *f*_e_ = 100 kHz, *l*_p_ = 0.8 *r_a_*; (**f**) *f*_e_ = 100 kHz, *l*_p_ = 1.6 *r_a_*.

In a mechanical system, numerous wear particles exist in the lubricating oil, so multiple particles may pass through the sensor simultaneously. To study the sensor responses under this condition, the flux density distribution of the dual wear particles system is analyzed based on the above model. The radii of wear particles are all set to *r_a_* = 0.125 mm, and the amplitude of background magnetic flux density is also set to 2.5 mT, and the interparticle distance *l*_p_ is set to 0.4 *r_a_*, 0.8 *r_a_*, 1.6 *r_a_*, and 2 *r_a_* respectively. Figure 8a–c illustrate the flux density distribution of the dual wear particles system with different distances under the static magnetic field, and (d)~(f) depict the flux density distribution distribution of the particles system under the alternating magnetic field with a frequency of 100 kHz.

The results show that when the dual wear particles pass through the sensor simultaneously, the magnetic flux density between the two wear particles is larger than the flux density of the outsides of wear particles. This phenomenon may because of the magnetic coupling effect between the dual wear particles. Under high frequency alternating magnetic field, the combination of the eddy current effect and the magnetic coupling effect make the magnetic field mainly distributed at the surface and between the two wear particles.

**Figure 9:**
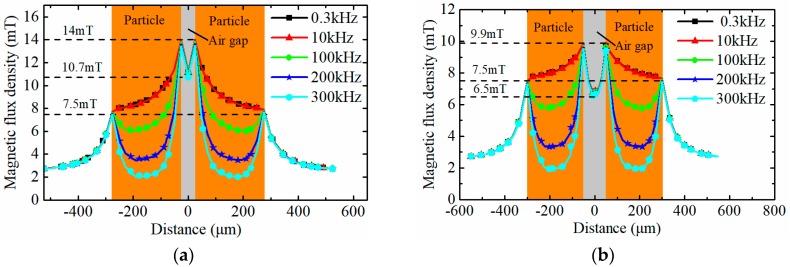

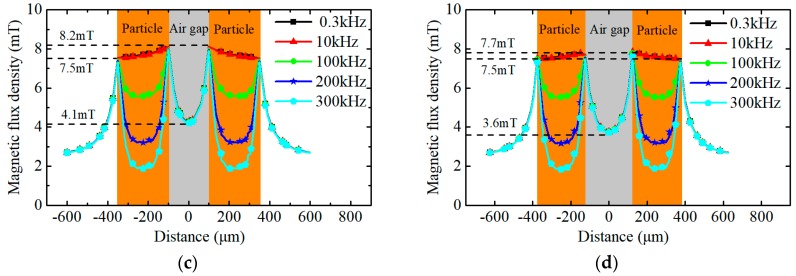
The flux density distribution along the *x* axis of the dual wear particles system (**a**) *l*_p_ = 0.4 *r_a_*; (**b**) *l*_p_ = 0.8 *r_a_*; (**c**) *l*_p_ = 1.6 *r_a_*; (**d**) *l*_p_ = 2.0 *r_a_*.

Figure 9 describes the flux density of the dual wear particles system with various interparticle distances along the *x* axis. The orange and gray area indicate the position of the wear particles and the air gap between the particles. As shown in the figure, under alternating magnetic field, the eddy current effect leads to the uneven magnetic distribution in the wear particles, and with the increase of the field frequency, the flux density in the center of the particles tends to 0. Meanwhile, compared with the magnetic properties of the single-particle model (Figure 5a), the magnetic flux density at the single-particle surface along the *x* axis is 7.48 mT, while the peak values of the magnetic flux density of the dual particles system along *x* axis with different interparticle distances reach to 14 mT (*l*_p_ = 0.4 *r_a_*), 9.9 mT (*l*_p_ = 0.8 *r_a_*), 8.2 mT (*l*_p_ = 1.6 *r_a_*), and 7.7 mT (*l*_p_ = 2 *r_a_*), respectively. The results clearly show that the magnetic coupling effect boosts the magnetic flux density in the airgap between the two particles, and with the increase of the interparticle distance, the magnetic coupling effect gradually declines and disappears.

**Figure 10:**
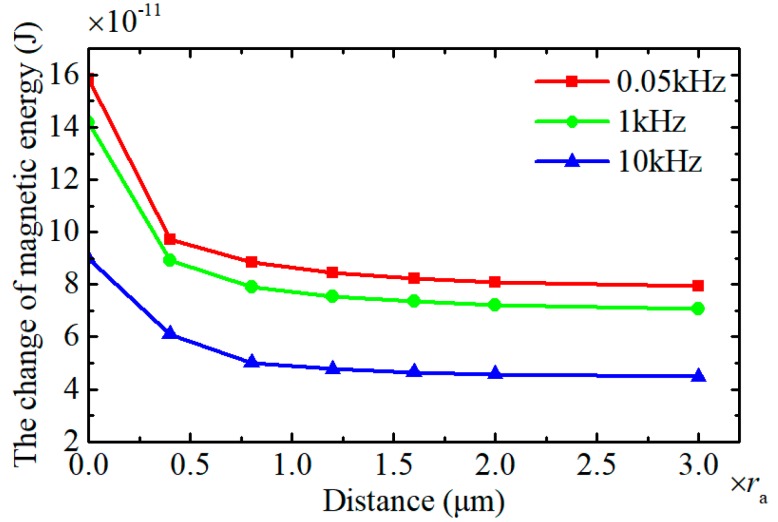
The change of the magnetic energy resulted from the dual wear particles system.

For the wear particles in the sensor, considering the hysteresis loss and eddy current loss and the magnetic coupling effect between the wear particles, the change of the magnetic energy stored in the excitation coil resulted from the dual wear particles system is displayed in Figure 10. The result shows that with the increase of the interparticle distance, the change of the magnetic energy gradually reduces to a stable value which equals to the total energy change caused by two independent wear particles. In this case, the magnetic coupling effect between the particles disappears completely. Meanwhile, with the increase of the effective field frequency, the change of the magnetic energy caused by the dual wear particles system declines.

### 3.4. The Sensor Signal Compensation

The ferromagnetic wear particles in the sensor change the flux density distribution of the sensor and the magnetic energy stored in the excitation coil, which lead to a rapid change of the magnetic flux through the induction coil and make the induction coil breed the induced electromotive force. Figure 7 and Figure 9 show that change of the magnetic energy caused by wear particles greadually decreases with the increase of the effective field frequency, which means that the induced electromotive force caused by certain wear particles also reduces with the increase of the effective field frequency.

For the calculation of the induced electromotive force output by the sensor, in traditional studies, the variation of magnetic flux through the excitation coil of the sensor caused by wear particles is estimated by the equation $\Delta \phi =N_{e}\iint \Delta Bds$, where *N*_e_ is the turns of the excitation coil, ΔB is the change of the flux density, and *s* is the cross sectional area of the induction coil. However, the change of the magnetic flux density caused by wear particles only occurs at the local position around the particle, and the magnetic leakage exists between the excitation coils and the induction coil. Therefore, the traditional calculation method over-estimates the magnetic flux variation of the induction coil, especially in the case of that the width of the excitation coil *a* is greatly larger than the diameter of the particle.

The modified calculation method of the total magnetic flux variation of the induction coil caused by wear particles can be expressed as:

(19) Δf=λ∑∬γΔBds

where $\lambda =\phi_{i}/\phi_{e}$ is the magnetic leakage ratio which is related to the structural and electric parameters of the sensor, $\phi_{i}$ and $\phi_{e}$ are the magnetic flux of induction coil and excitation coil in a steady-state, and $\gamma =N_{e}/a$ is the linear density of the excitation coil turns.

**Figure 11:**
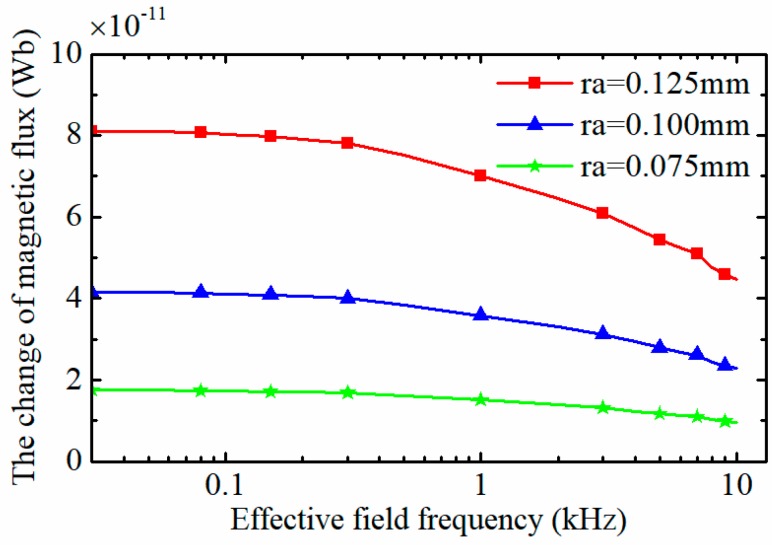
The change of the magnetic flux caused by wear debris under different effective field frequency.

Through the calculation by the finite element method, the magnetic leakage ratio λ=0.201 under the sensor parameters of *N*_e_ = 135, *N*_i_ = 95, *I*_0_ = 0.24A, *a* = 2 mm, and *m* = 9 mm. The changes of magnetic flux through the induction coil of the sensor caused by wear particles are shown in Figure 11.

It can be seen that with the increase of the effective field frequency, the change of magnetic flux through the induction coil declines gradually. For the three-coil particle detection sensor as shown Figure 1a, the induced electromotive force output by the sensor *E*_o_ can be expressed as:

(20) $$E_{o}=K\lambda N_{i}\frac{\Delta \phi }{\Delta t}=K\lambda \omega N_{i}\phi_{m}(f_{e},V_{p})\sin(\omega t)$$

where *K* = 100 is the signal gain factor of the measureent system, *N*_i_ is the turns of the induction coil, $\omega =2\pi f_{0}$ is the angle frequency, $f_{0}=100\;\mathrm{kHz}$ is the excitation frequency of the sensor, *f*_e_ is the effective field frequency, *V*_p_ is the volume of the wear particle, and $\phi_{m}$ is the amplitude of the magnetic flux through the induction coil.

For the wear particles detection sensor with certain structural and circuit parameters, the induced electromotive force depends on $\phi_{m}$ which varies with particle volume and effective field frequency. When a wear particle move through the sensor with different speeds, the effective field frequency changes, which indirectly leads to the variation of the induced electromotive force output by the sensor. To eliminate the influence on the induced electromotive force by particle speed and improve the consistency of the detection results, the signal compensation method is presented based on the above physical model.

**Figure 12:**
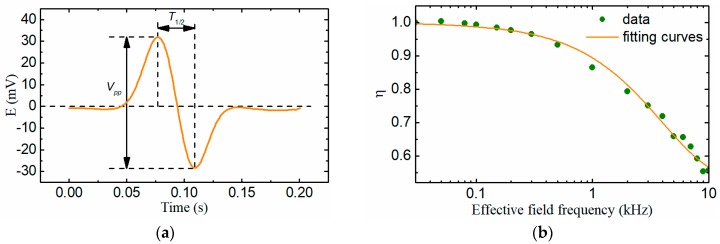
(**a**) A typical particle signal output by the sensor; (**b**) The relative change ratio of the magnetic flux.

A typical particle signal output by the sensor is shown in Figure 12a from which the peak-peak value *V*_pp_ and the half a period *T*_1/2_ of the induced electromotive force can be extracted. In the detection process of the wear particles, the peak-peak value is used to estimate the size of the particle and the half a period reflects the particle speed as *v* = *l*/(2*T*_1/2_). The effective field frequency can be calculated as *f*_e_ = *f*_0_*2*T*_1/2_.

Based on the data of the Figure 11, the relative change ratio of the magnetic flux through the induction coil caused by wear particles of different sizes under various effective field frequencies is calculated and displayed in Figure 12b, which means that when the effective field frequency is 10 kHz (the corresponding particle speed is 0.12 m/s), the induced electromotive force reduces by about 45%. The relative change ratio of the magnetic flux η was characterized by the exponential curve fitting method as Equation (21):

(21) $$\eta (f_{e})=0.52855+0.47023e^{-\frac{f_{e}}{39.716}}$$

The induced electromotive force output by the sensor should be revised by Equation (22) to counteract the hysteresis loss and eddy current loss of the particle under different effective field frequencies(or particle speeds) and improve the consistency of the detection result:

(22) $$E_{e}=\frac{E_{o}}{\eta (f_{e})}=\frac{V_{\mathrm{pp}}}{\eta (f_{0}\times 2T_{1/2})}$$

## 4. Experiments

The magnetic property model of wear particles demonstrates that the ferromagnetic particles increase the magnetic energy of the local magnetic field. Under the alternating magnetic field, considering the hysteresis effect and the eddy current effect of the particles, the change of the magnetic energy declines with the increase of the effective field frequency. For the dual wear particles system, the magnetic coupling effect greatly enhances the change of the magnetic energy and gradually weakens with the rise of the interparticle distance. The change of the magnetic energy caused by wear particles can be reflected by the induced electromotive force output by the wear particles detection sensor.

**Figure 13:**
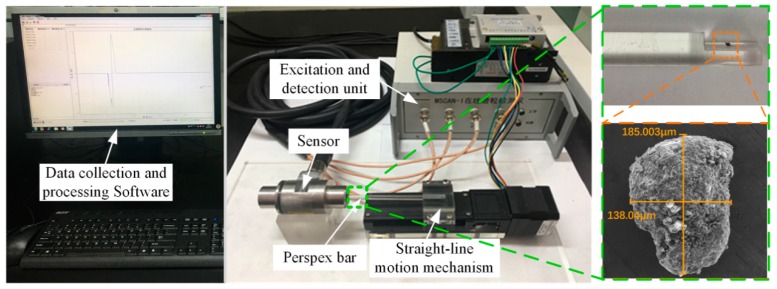
The experiment system.

The experimental system, which includes the data collection and processing software, excitation and detection unit, the sensor, and a straight-line motion machinery, is shown in Figure 13. The detailed parameters of the experiment system are listed in Table 2. In the experiments, to guarantee the wear particles always move along the axis of the sensor, the tested wear particles were stuck on the center plane of a Perspex bar whose diameter is same as the aperture of the sensor. The straight-line motion machinery drives the Perspex bar to reciprocate through the sensor. The excitation and detection unit generates the sinusoidal excitation currents for the sensor and extracts the weak induced electromotive forces. Finally, the particles signal is displayed in the data collection and processing software.

### 4.1. The Sensor Responses Caused by Single Wear Particle

**Figure 14:**
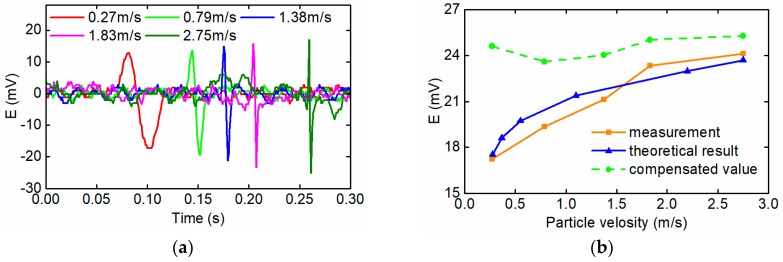
(**a**) Induced electromotive force caused by a wear particle with different speeds; (**b**) Comparison analysis between measurement and theoretical results.

To validate the variation rule of the magnetic energy caused by the wear particle under alternating magnetic field with different frequencies, the output electromotive forces of the sensor, when a wear particle with diameter of 180 μm passes through the sensor with different speeds, are measured and shown in Figure 14a. In the mechanical equipments, the maximum flow rate of the lubricating oil is 4 m/s, while in the experiment, the limitation of the sampling rate of the excitation and detection unit makes the sensor can’t detect the wear particles with speed higher than 3 m/s.

The experiment results show that with the increase of the particle speeds from 0.27 m/s to 2.75 m/s, the magnitude of the output electromotive force of the sensor also rises from 30 mV to 42 mV. Although the excitation frequency of the sensor is a constant, the effective field frequency changes with the variation of the particle speeds. Therefore, the hysteresis loss and the eddy current loss are varying with the effective field frequencies, since they have a positive relationship to the effective field frequencies. The varying hysteresis loss and the eddy current loss further influence the distribution and the magnitude of the flux density, then the output electromotive forces of the sensor change.

The comparison between the measured results and the calculated results by the proposed models is depicted in the Figure 14b. The blue curve represents the theoretical induced electromotive force based on the magnetic properties model of the signal wear particle as the Section 3.2 and the orange curve is the measurement results in the experiment. It can be seen that the theoretical results are commendably consistent with the experimental data. Both the measured results and the calculated results by the proposed modes are proportional to the speeds of the wear particle, and the error between them is less than 5%.

In order to obtain a unified particle signal when the same wear particle passing through the sensor in different speeds, the signal compensation method is introduced in this system. The raw induced electromotive force output by the sensor should be revised by the Equation (22). The modified particle signals is given by the green curve in Figure 14b, which shows that after the signal compensation, the consistency of the particle signal is significantly improved.

### 4.2. The Sensor Responses Caused by Dual Wear Particles System

**Figure 15:**
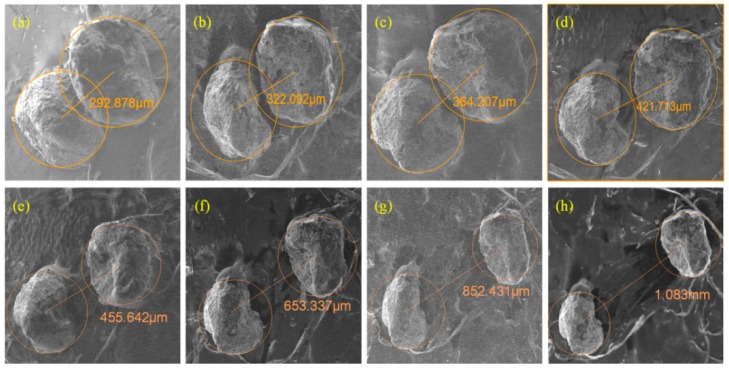
The dual particle system at different distances. (**a**) *l*_p_ = 292.878 μm (overlapping wear particles); (**b**) *l*_p_ = 322.092 μm; (**c**) *l*_p_ = 364.207 μm; (**d**) *l*_p_ = 421.713 μm; (**e**) *l*_p_ = 455.642 μm; (**f**) *l*_p_ = 653.337 μm; (**g**) *l*_p_ = 852.431 μm; (**h**) *l*_p_ = 1.083 mm.

**Figure 16:**
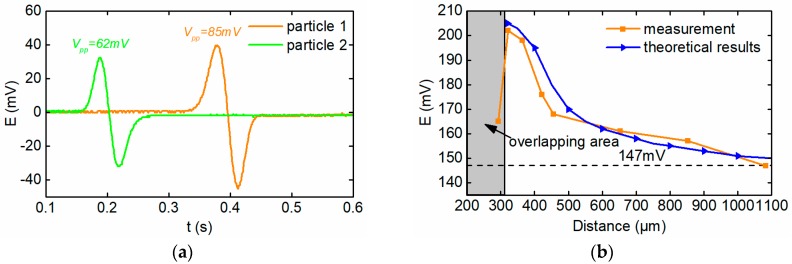
The signals. (**a**) The signal of single particle; (**b**) the signal of dual particle system.

To verify the magnetic coupling effect between the adjacent particles in the sensor, two wear particles with diameters of 280 μm and 340 μm are stuck on the Perspex bar at different distances as shown in Figure 15. Figure 15a shows the two overlapping wear particles, and (b)–(h) show the wear particles with different interparticle distances from 322 μm~1 mm. The line-motion machinery is used to drive the two wear particles to pass through the sensor simultaneously. As a comparison, firstly, let these two particles pass through the sensor separately at the speed of 0.1 m/s, the output electromotive forces of the sensor are 62 mV and 85 mV respectively, as shown in Figure 16a. In this situation, the sum of the output electromotive forces of the sensor is 147 mV.

When the coupled wear particles pass through the sensor simultaneously, the magnitudes of the sensor’s output electromotive force are large than 147 mV. The comparison between the measured results and the theoretical results is depicted in the Figure 16b, which shows that the magnetic coupling effect between two adjacent wear particles(non-overlapping) enhances the induced electromotive force output by the sensor. When there is a small gap between the two wear particles as Figure 15b, the induced electromotive force of the sensor is 202 mV which is much larger than the sum of signals of the two independent wear particles (147 mV). With further increase of the interparticle distance, the magnetic coupling effect gradually recedes and the sensor output signal gradually decreases to 147 mV, which means that the magnetic coupling effect disappears completely.

There is a specific case that when the coupled wear particles have some overlapping areas as Figure 15a, the induced electromotive force of the sensor (165 mV) is much lower than signal under the situation that there is a tiny airgap between the wear particles as Figure 15b (202 mV). This may result from that the distribution of magnetic field of the overlapping wear particles system tends to be continuous and the magnetic coupling effect is relatively weak.

## 5. Conclusions

This paper builds the magnetic property models of the wear particles under an alternating magnetic field to precisely estimate the change of magnetic energy of the sensor coil caused by the wear particles. These models consider the eddy current effect and hysteresis effect of the ferromagnetic wear particles under the alternating magnetic field and analyse the influence on the change of the magnetic energy by the effective field frequency. Meanwhile, the magnetic coupling effect between two adjacent wear particles is studied. Based on this work, the following conclusions are obtained:

(1) The change of the magnetic energy of the coil caused by wear particles is composed of the energy change in the wear particle and the surrounding air. Meanwhile, because the exist of the hysteresis loss and eddy current loss, the change of magnetic energy decreases with the increase of the effective field frequency.
(2) When two adjacent wear particles pass through the sensor simultaneously, the magnetic coupling effect between the two particles enhances the change of the magnetic enenrgy of the sensor coil, which make the induced electromotive force output by the sensor is larger than the sum of signals of the two independent wear particles.
(3) For two overlapping wear particles, the magnetic coupling effect is relatively weak. While once a tiny air gap exists between the two wear particles, the magnetic coupling effect enhances rapidly, and with the increase of the interparticle distances, the magnetic coupling effect gradually reduces and disappears.
(4) The particle speed changes the effective field frequency and then affects the induced electromotive force output by the sensor. The lower particle speed increases the magnetic energy loss, which reduces the particle signal and vice versa. Therefore, the signal compensation method is needed to obtain a unified particle signal when the same wear particle passing through the sensor in different speeds.

## Figures and Tables

**Table 1:** The relative complex permeability of material under different field frequencies.

Field Frequency (kHz)	Relative Complex Permeability
0.05	45.689-37.968i
0.3	19.450-18.518i
1	11.083-10.045i
2	7.159-4.958i
4	5.8156-3.2477i
6	5.1825-2.0928i
8	4.3601-1.4846i
10	3.9945-1.2478i

**Table 2:** The detailed parameters of the experiment system.

Parameters	Value
Inner diameter of the sensor *d* (mm)	7
Inner radius of the coils *R*_1_ (mm)	10
Width of coils *a* (mm)	2
Center distance between the excitation coils *m* (mm)	9
Outer distance between the excitation coils *l* (mm)	11
Turns of excitation coil *N*_e_	127
Turns of induction coil *N*_i_	90
Excitation current *I*_0_ (A)	0.24sin(2π*f*_0_*t*)
Excitation frequency *f*_0_ (kHz)	100
Signal gain factor of the measurement system *K*	100
